# Increased health service use for asthma, but decreased for COPD: Northumbrian hospital episodes, 2013–2014

**DOI:** 10.1007/s10096-015-2547-y

**Published:** 2016-01-15

**Authors:** I. Shiue

**Affiliations:** Northumbria Healthcare NHS Foundation Trust, Newcastle upon Tyne, UK; Department of Healthcare, Northumbria University, Newcastle upon Tyne, NE1 8ST England UK

## Abstract

The burden of respiratory disease has persisted over the years, for both men and women. The aim of the present study was to investigate the hospital episode rates in respiratory disease and to understand whether and how the use of the health service for respiratory disease might have changed in recent years in the North-East of England. Hospital episode data covering two full calendar years (in 2013–2014) was extracted from the Northumbria Healthcare NHS Foundation Trust, which serves a population of nearly half a million. Hospital episode rates were calculated from admissions divided by annual and small area-specific population size by sex and across age groups, presented with per 100,000 person-years. The use of the health service for influenza and pneumonia, acute lower respiratory infections and chronic obstructive pulmonary disease (COPD) increased with an advancing age, except for acute upper respiratory infections and asthma. Overall, the use of the health service for common respiratory diseases has seemed to be unchanged, except for asthma. There were large increases in young adults aged 20–50 for both men and women and the very old aged 90+ in women. Of note, there were large increases in acute lower respiratory infections for both men and women aged 90+, whereas there was also a large decrease in COPD in women aged 80–90. This is the first study to examine health service use for respiratory diseases by calculating the detailed population size as denominator. Re-diverting funding to improve population health on a yearly basis may serve the changing need in local areas.

## Introduction

### Evidence before this study

Respiratory disease, as an adult health condition, affects millions of people globally and is the one of the leading causes of health issues in both developed and developing countries [1]. Health service use has increased in older persons and costs millions of pounds in the UK, USA and several European countries, which could prompt considerations on long-term healthcare together with the entire socio-economic structure [2–5]. Hospital admissions have seemed to decrease in some regions, whereas in other regions primary care consultations seem to have increased, likely due to different study populations, study time periods and/or estimation methods in rates [6–28]. Continuously monitoring how people consume the health service because of various health conditions is important in assisting with individual, local and national health profiles and with the re-allocation of medical and social recourse effectively and consequently to prevent from unnecessary pain and spending. Therefore, such clinical evidence is necessary.

### Knowledge gap

Investigating admission rates and hospitalisation rates could be perceived as a direct way of understanding how many patients are admitted and hospitalised require health service utilisation. Previous research tended to estimate age-standardised rates using the population census in a certain year by accommodating a specific population structure (e.g. Europe) or by adjusting for all ages in a specific study catchment to compare across countries and/or regions. However, looking at the total age-standardised rate by using the population census in a certain year may sometimes mislead and misguide the re-allocation of local medical and social resources, as one national, international or global policy does not always fit all owing to different unadjusted historical contexts (i.e. biological or non-biological risk contributor profiles).

### Study aim

Following this context, therefore, the aim of the present study was to investigate the age-specific hospital episode rates in common respiratory diseases by sex and across age groups using an annual and small area-specific population size to understand and establish the monitoring on whether and how the use of the health service for respiratory diseases may have changed in recent years, if at all.

## Materials and methods

### Study sample

Hospital Episode Statistics (HES; more details via http://www.hscic.gov.uk/hes) is a data warehouse containing details of all admissions, outpatient appointments and A&E attendances at National Health Service (NHS) hospitals in England. These data are collected during a patient's time at hospital and are submitted to allow hospitals to be paid for the care they deliver. HES data are designed to enable secondary use, particularly for non-clinical purposes. Each NHS trust in England collects its own patient data, and the anonymised data are kept locally within each trust and also centrally at the national level. Northumbria Healthcare NHS Foundation Trust (more details via https://www.northumbria.nhs.uk/) covers the health service mostly for Northumberland and North Tyneside, including three major hospitals (Hexham General Hospital, North Tyneside General Hospital and Wansbeck General Hospital) and other smaller community hospitals (Alnwick Infirmary, Berwick Infirmary, Blyth Community Hospital, Haltwhistle War Memorial Hospital, Rothbury Community Hospital and Sir G B Hunter Memorial Hospital) facilitating health and social care and well-being for rehabilitation purposes (more details via http://www.nhs.uk/Services/Trusts/Overview/DefaultView.aspx?id=1802) and acts as a foundation trust that has been free from central government control since 2006 (more details via https://www.northumbria.nhs.uk/about-us/being-foundation-trust).

### Variables and analyses

The data from the Northumbrian Hospital Episodes used in the present study covered two full calendar years (2013–2014). Health service use was determined by each admission coded as J00-06 Acute upper respiratory infections, J09-18 Influenza and pneumonia, J20-J22 Acute lower respiratory infections, G44 Other chronic obstructive pulmonary disease (COPD) and J45 Asthma, based on the International Classification of Diseases, 10th version (more details via http://apps.who.int/classifications/icd10/browse/2015/en; now re-directed to http://apps.who.int/classifications/icd10/browse/2016/en). To estimate the usage of the health service, age-specific HES rates were calculated from admissions divided by population size for each age group, presented with per 100,000 person-years. Estimates on population size in both 2013 and 2014 were obtained from the UK Office for National Statistics (more details via http://www.ons.gov.uk/ons/taxonomy/index.html?nscl=Population). Statistical software STATA version 13.0 (STATA, College Station, Texas, USA; more details via http://www.stata.com/) and Microsoft Excel (more details via https://products.office.com/en-us/excel) were used to perform all the analyses and to generate graphs. As this was only a secondary data analysis with no individual identification in the present study, no further ethics approval was required.

## Results

**Fig. 1:**
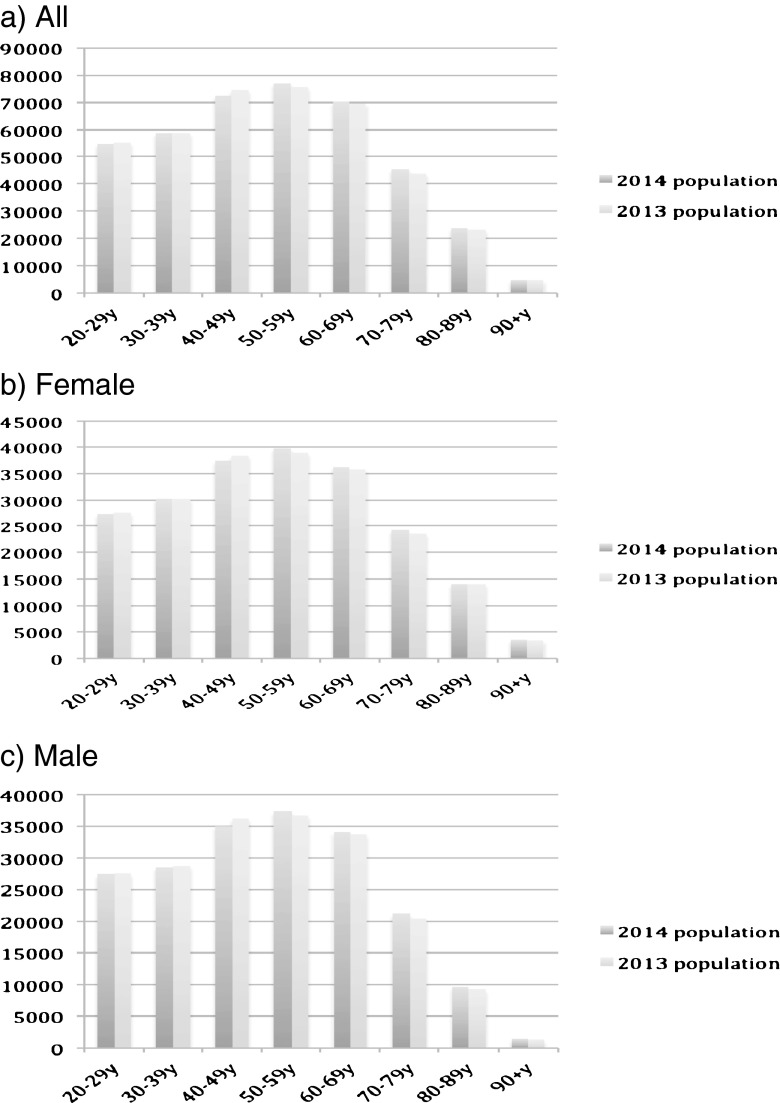
Population size by sex and across age groups in Northumbria

**Fig. 2:**
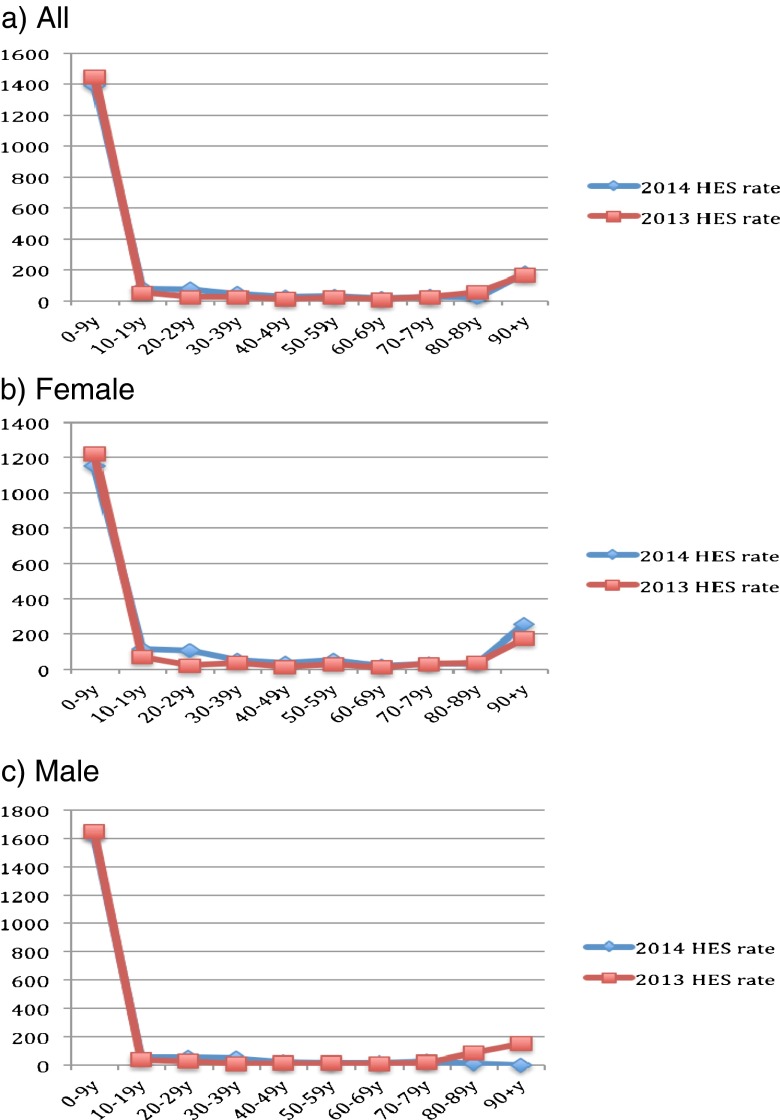
Distribution of rates of health service use for “J00–J06: acute upper respiratory infections”

**Fig. 3:**
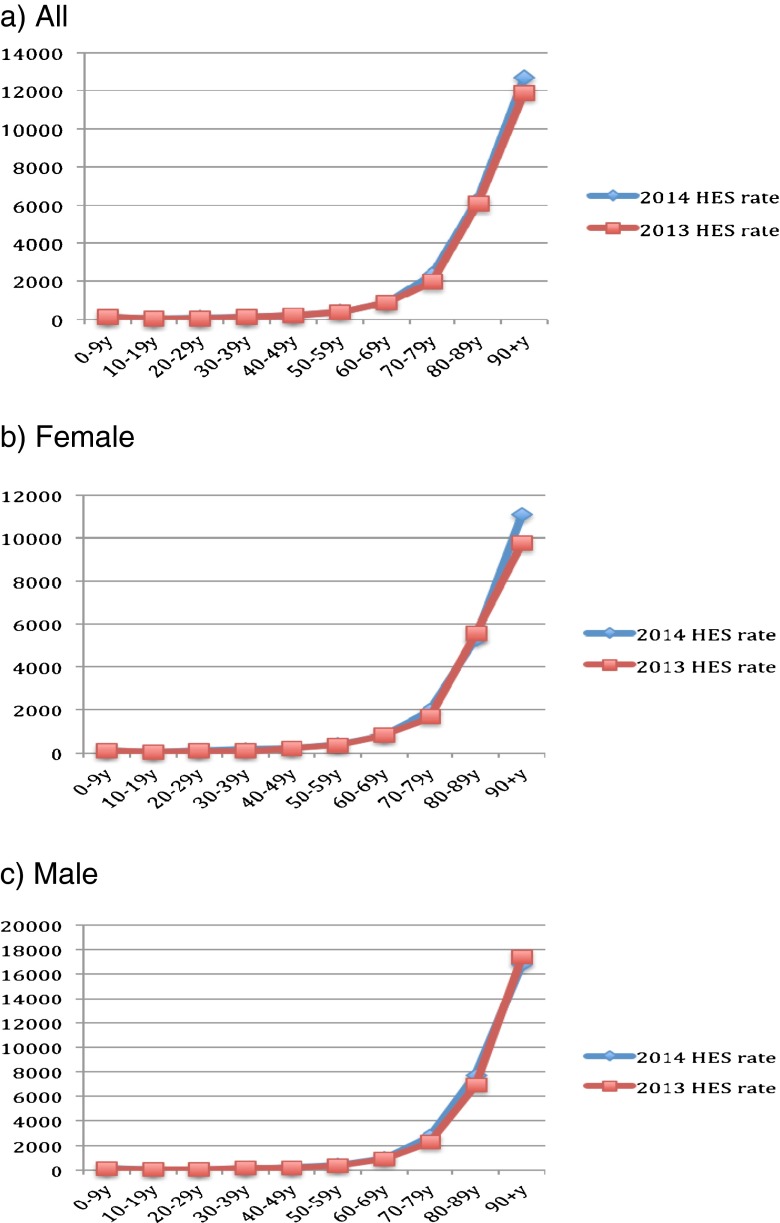
Distribution of rates of health service use for “J09–J18: influenza and pneumonia”

**Fig. 4:**
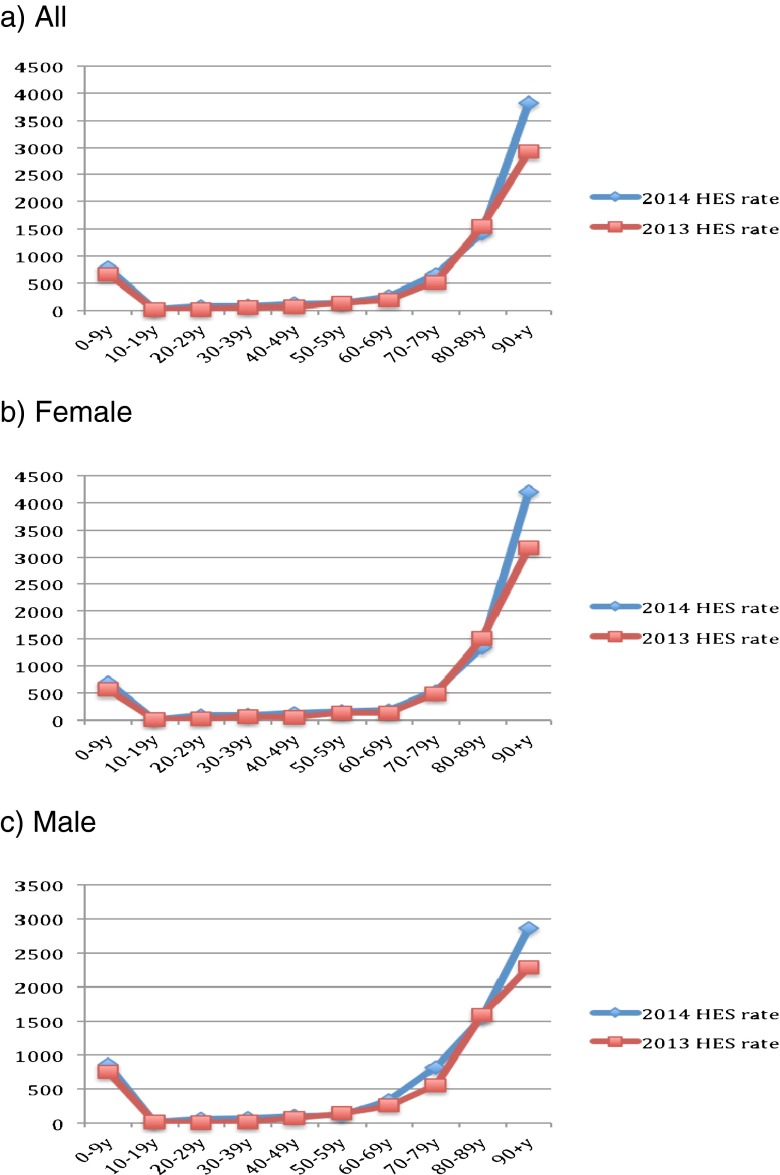
Distribution of rates in health service use for “J20–J22: other acute lower respiratory infections”

**Fig. 5:**
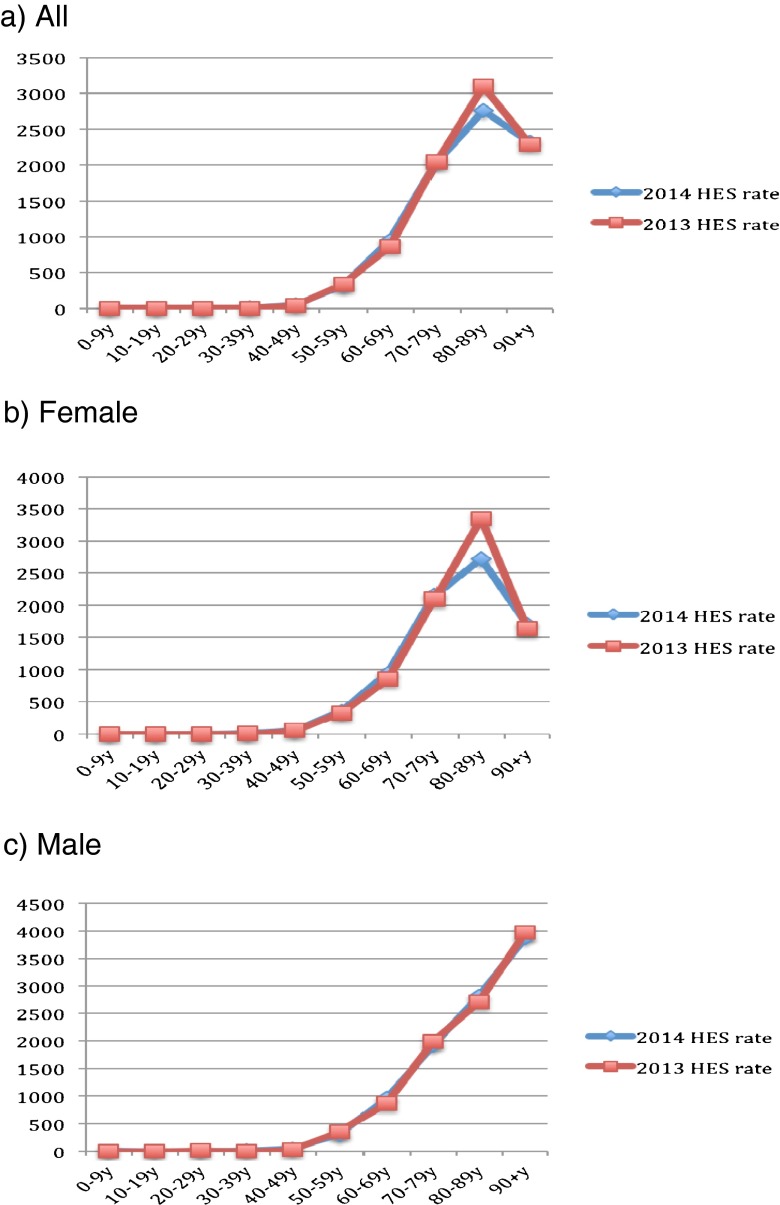
Distribution of rates of health service use for “J44: COPD” (chronic obstructive pulmonary disease)

**Fig. 6:**
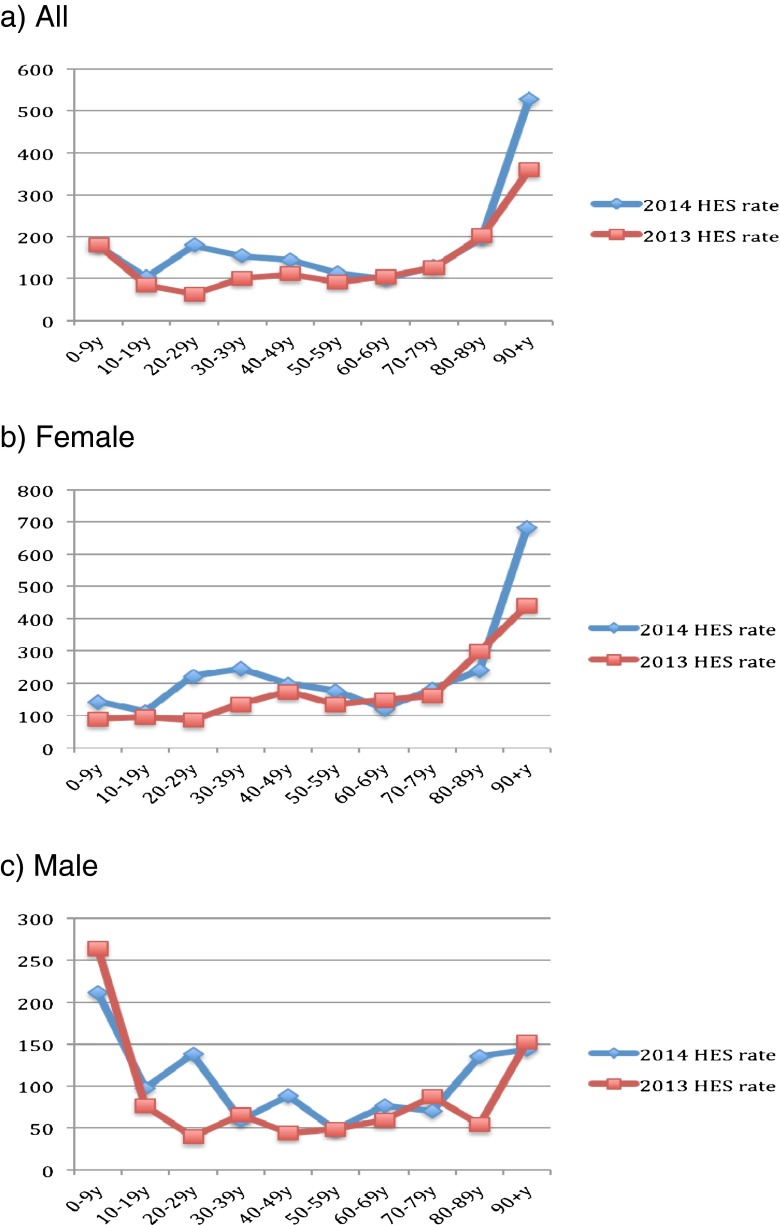
Distribution of rates of health service use for “J45: asthma”

Figure 1 describes the population size by sex and across age groups in mid-2013 to mid-2014. Clearly, the population of young adults (aged 20–49) has decreased, whereas that of older adults (aged 50 and above) has increased. Figures 2–6 show the distribution of rates of health service use for acute upper respiratory infections, influenza and pneumonia, acute lower respiratory infections, COPD and asthma from 2013 to 2014 by sex and age groups respectively (also see Tables 1–5). Clearly, the use of the health service for influenza and pneumonia, acute lower respiratory infections and COPD increased with an advancing age in both men and women, but not for acute upper respiratory infections and asthma. Following these 2 years, the use of the health service for common respiratory diseases has seemed to be unchanged, except for asthma. There were large increases in young adults aged 20–50 for both men and women and the very old aged 90 and above in women. Of note, there were large increases in acute lower respiratory infections for both men and women aged 90 and above; there was also a large decrease in COPD in women aged 80–90.

**Table 1:** Hospital episode statistics for “J00–J06: acute upper respiratory infections”

2014				2013			
All (years)	Episode	Population	2014 HES rate	All age groups (years)	Episode	Population	2013 HES rate
0–9	775	55,577	1394.461738	0–9	802	55,550	1443.744374
10–19	47	55,577	84.567357	10–19	30	56,221	53.36084381
20–29	44	54,879	80.17638805	20–29	14	55,221	25.3526738
30–39	30	58,734	51.07774032	30–39	14	58,955	23.74692562
40–49	21	72,433	28.99231013	40–49	10	74,655	13.3949501
50–59	27	77,070	35.0330868	50–59	16	75,724	21.12936453
60–69	13	70,296	18.49322863	60–69	7	69,558	10.06354409
70–79	14	45,482	30.78140803	70–79	11	44,044	24.97502498
80–89	6	23,764	25.2482747	80–89	13	23,324	55.73658035
90+	9	4,919	182.9640171	90+	8	4,716	169.6352841
Total	164	40,7577	40.23779556	Total	93	406,197	22.89529465
Female (years)							
0–9	309	26,728	1156.090991	0–9	327	26767	1221.653529
10–19	32	26,938	118.7912985	10–19	19	27247	69.73244761
20–29	30	27,406	109.4650806	20–29	6	27663	21.68962152
30–39	17	30,170	56.34736493	30–39	11	30200	36.42384106
40–49	14	37,372	37.4612009	40–49	5	38432	13.00999167
50–59	22	39,723	55.38353095	50–59	11	38943	28.24641142
60–69	7	36,233	19.31940496	60–69	4	35817	11.16788117
70–79	8	24,226	33.02237266	70–79	7	23546	29.72904103
80–89	5	14,148	35.3406842	80–89	5	14045	35.5998576
90+	9	3,525	255.3191489	90+	6	3407	176.1080129
Total	112	212,803	52.63083697	Total	55	212053	25.936912
Male (years)							
0–9	466	28,849	1615.30729	0–9	475	28,783	1650.279679
10–19	15	28,609	52.43105317	10–19	11	28,558	38.51810351
20–29	14	27,473	50.9591235	20–29	8	27,558	29.02968285
30–39	13	28,564	45.51183308	30–39	3	28,755	10.43296818
40–49	7	35,061	19.9652035	40–49	5	36,223	13.80338459
50–59	5	37,347	13.38795619	50–59	5	36,781	13.59397515
60–69	6	34,063	17.61442034	60–69	3	33,741	8.891259892
70–79	6	21,256	28.22732405	70–79	4	20,498	19.51409894
80–89	1	9,616	10.39933444	80–89	8	9,279	86.21618709
90+	0	1,394	0	90+	2	1,309	152.7883881
Total	52	194,774	26.69760851	Total	38	194,144	19.57310038

**Table 2:** Hospital episode statistics for “J09–J18: influenza and pneumonia”

2014				2013			
All (years)	Episode	Population	2014 HES rate	All age groups (years)	Episode	Population	2013 HES rate
0–9	67	55,577	120.5534664	0–9	66	55,550	118.8118812
10–19	26	55,577	46.78194217	10–19	16	56,221	28.4591167
20–29	41	54,879	74.70981614	20–29	31	55,221	56.13806342
30–39	73	58,734	124.2891681	30–39	75	58,955	127.215673
40–49	147	72,433	202.9461709	40–49	147	74,655	196.9057665
50–59	312	77,070	404.8267808	50–59	272	75,724	359.1991971
60–69	620	70,296	881.9847502	60–69	600	69,558	862.5894937
70–79	1,069	45,482	2,350.38037	70–79	868	44,044	1,970.756516
80–89	1,494	23,764	6,286.820401	80–89	1,420	23,324	6,088.149546
90+	625	4,919	12,705.83452	90+	561	4,716	11,895.6743
Total	4,474	407,577	1,097.706691	Total	4,056	406, 197	998.5302698
Female (years)							
0–9	28	26,728	104.7590542	0–9	27	26,767	100.8704748
10–19	10	26,938	37.12228079	10–19	9	27,247	33.03115939
20–29	25	27,406	91.22090053	20–29	24	27,663	86.75848606
30–39	46	30,170	152.4693404	30–39	29	30,200	96.02649007
40–49	76	37,372	203.3608049	40–49	80	38,432	208.1598668
50–59	156	39,723	392.7195831	50–59	141	38,943	362.0676373
60–69	300	36,233	827.9744984	60–69	300	35,817	837.591088
70–79	482	24,226	1,989.597953	70–79	398	23,546	1,690.308333
80–89	750	14,148	5,301.102629	80–89	780	14,045	5,553.577786
90+	391	3,525	11,092.19858	90+	333	3,407	9,773.994717
Total	2,264	212,803	1,063.894776	Total	2,121	212,053	1,000.221643
Male (years)							
0–9	39	28,849	135.1866616	0–9	39	28,783	135.4966473
10–19	16	28,609	55.92645671	10–19	7	28,558	24.51152041
20–29	16	27,473	58.23899829	20–29	7	27,558	25.40097249
30–39	27	28,564	94.52457639	30–39	46	28,755	159.9721788
40–49	71	35,061	202.504207	40–49	67	36,223	184.9653535
50–59	156	37,347	417.7042333	50–59	131	36,781	356.1621489
60–69	320	34,063	939.4357514	60–69	300	33,741	889.1259892
70–79	587	21,256	2,761.573203	70–79	470	20,498	2,292.906625
80–89	744	9,616	7,737.104825	80–89	640	9,279	6,897.294967
90+	234	1,394	16,786.22669	90+	228	1,309	17,417.87624
Total	2,210	194,774	1,134.648362	total	1,935	194,144	996.6828746

**Table 3:** Hospital episode statistics for “J20–J22: other acute lower respiratory infections”

2014				2013			
All (years)	Episode	Population	2014 HES rate	All age groups (years)	Episode	Population	2013 HES rate
0–9	436	55,577	784.4971841	0–9	372	55,550	669.6669667
10–19	10	55,577	17.99305468	10–19	9	56,221	16.00825314
20–29	40	54,879	72.8876255	20–29	10	55,221	18.10905272
30–39	48	58,734	81.72438451	30–39	28	58,955	47.49385124
40–49	83	72,433	114.5886543	40–49	49	74,655	65.63525551
50–59	105	77,070	136.239782	50–59	102	75,724	134.6996989
60–69	180	70,296	256.0600888	60–69	134	69,558	192.6449869
70–79	304	45,482	668.3962886	70–79	229	44,044	519.9346108
80–89	339	23,764	1,426.527521	80–89	359	23,324	1,539.187103
90+	188	4,919	3,821.915023	90+	138	4,716	2,926.208651
Total	1,733	407,577	425.1957299	Total	1,430	406,197	352.0459285
Female (years)							
0–9	186	26,728	695.8994313	0–9	153	26,767	571.5993574
10–19	3	26,938	11.13668424	10–19	3	27,247	11.01038646
20–29	23	27,406	83.92322849	20–29	8	27,663	28.91949535
30–39	27	30,170	89.49287372	30–39	21	30,200	69.53642384
40–49	46	37,372	123.086803	40–49	22	38,432	57.24396336
50–59	63	39,723	158.5982932	50–59	50	38,943	128.3927792
60–69	65	36,233	179.3944747	60–69	46	35,817	128.4306335
70–79	129	24,226	532.4857591	70–79	114	23,546	484.1586681
80–89	190	14,148	1,342.945999	80–89	211	14,045	1,502.313991
90+	148	3,525	4,198.58156	90+	108	3,407	3,169.944232
Total	880	212,803	413.5280048	Total	736	212,053	347.0830406
Male (years)							
0–9	250	28,849	866.581164	0–9	219	28,783	760.8657888
10–19	7	28,609	24.46782481	10–19	6	28,558	21.00987464
20–29	17	27,473	61.87893568	20–29	2	27,558	7.257420713
30–39	21	28,564	73.51911497	30–39	7	28,755	24.34359242
40–49	37	35,061	105.5303614	40–49	27	36,223	74.53827679
50–59	42	37,347	112.458832	50–59	52	36,781	141.3773416
60–69	115	34,063	337.6097232	60–69	88	33,741	260.8102902
70–79	175	21,256	823.2969514	70–79	115	20,498	561.0303444
80–89	149	9,616	1,549.500832	80–89	148	9,279	1,594.999461
90+	40	1,394	2,869.440459	90+	30	1,309	2,291.825821
Total	853	194,774	437.9434627	Total	694	194,144	357.4666227

**Table 4:** Hospital episode statistics for “J44: COPD” (chronic obstructive pulmonary disease)

2014				2013			
All (years)	Episode	Population	2014 HES rate	All	Episode	Population	2013 HES rate
0–9	1	55,577	1.799305468	0–9	0	55,550	0
10–19	0	55,577	0	10–19	0	56,221	0
20–29	0	54,879	0	20–29	2	55,221	3.621810543
30–39	6	58,734	10.21554806	30–39	1	58,955	1.696208973
40–49	35	72,433	48.32051689	40–49	30	74,655	40.18485031
50–59	245	77,070	317.8928247	50–59	255	75,724	336.7492473
60–69	670	70,296	953.1125526	60–69	598	69,558	859.7141953
70–79	930	45,482	2,044.764962	70–79	902	44,044	2,047.952048
80–89	656	23,764	2,760.478034	80–89	722	23,324	3,095.523924
90+	114	4,919	2,317.544216	90+	108	4,716	2,290.076336
Total	2,657	407,577	651.9013585	Total	2,618	406,197	644.5148536
Female (years)							
0–9	0	26,728	0	0–9	0	26,767	0
10–19	0	26,938	0	10–19	0	27,247	0
20–29	0	27,406	0	20–29	0	27,663	0
30–39	4	30,170	13.25820351	30–39	2	30,200	6.622516556
40–49	20	37,372	53.51600128	40–49	21	38,432	54.64196503
50–59	136	39,723	342.3709186	50–59	127	38,943	326.1176591
60–69	342	36,233	943.8909282	60–69	304	35,817	848.7589692
70–79	521	24,226	2,150.582019	70–79	493	23,546	2,093.773889
80–89	385	14,148	2,721.232683	80–89	471	14,045	3,353.506586
90+	60	3,525	1,702.12766	90+	56	3,407	1,643.674787
Total	1,468	212,803	689.8398989	Total	1,474	212,053	695.1092416
Male (years)							
0–9	1	28,849	3.466324656	0–9	0	28,783	0
10–19	0	28,609	0	10–19	0	28,558	0
20–29	0	27,473	0	20–29	2	27,558	7.257420713
30–39	2	28,564	7.001820473	30–39	0	28,755	0
40–49	15	35,061	42.78257893	40–49	9	36,223	24.84609226
50–59	109	37,347	291.857445	50–59	128	36,781	348.0057638
60–69	328	34,063	962.9216452	60–69	294	33,741	871.3434694
70–79	409	21,256	1924.162589	70–79	409	20,498	1,995.316616
80–89	271	9,616	2818.219634	80–89	251	9,279	2,705.03287
90+	54	1,394	3873.74462	90+	52	1,309	3,972.49809
Total	1,189	194,774	610.4510869	Total	1,145	194,144	589.7684193

**Table 5:** Hospital episode statistics for “J45: asthma”

2014				2013			
All (years)	Episode	Population	2014 HES rate	All	Episode	Population	2013 HES rate
0–9	99	55,577	178.1312413	0–9	100	55,550	180.0180018
10–19	58	55,577	104.3597171	10–19	48	56,221	85.3773501
20–29	99	54,879	180.3968731	20–29	35	55,221	63.3816845
30–39	91	58,734	154.9358123	30–39	60	58,955	101.7725384
40–49	105	72,433	144.9615507	40–49	83	74,655	111.1780859
50–59	88	77,070	114.1819125	50–59	70	75,724	92.44096984
60–69	70	70,296	99.57892341	60–69	73	69,558	104.9483884
70–79	59	45,482	129.7216481	70–79	56	44,044	127.1455817
80–89	47	23,764	197.7781518	80–89	47	23,324	201.5091751
90+	26	4,919	528.562716	90+	17	4,716	360.4749788
Total	742	407,577	182.0514897	Total	589	406,197	145.0035328
Female (years)							
0–9	38	26,728	142.1730021	0–9	24	26,767	89.6626443
10–19	30	26,938	111.3668424	10–19	26	27,247	95.42334936
20–29	61	27,406	222.5789973	20–29	24	27,663	86.75848606
30–39	74	30,170	245.276765	30–39	41	30,200	135.7615894
40–49	74	37,372	198.0092048	40–49	67	38,432	174.3338884
50–59	70	39,723	176.2203258	50–59	52	38,943	133.5284904
60–69	44	36,233	121.4362598	60–69	53	35,817	147.9744256
70–79	44	24,226	181.6230496	70–79	38	23,546	161.3862227
80–89	34	14,148	240.3166525	80–89	42	14,045	299.0388038
90+	24	3,525	680.8510638	90+	15	3,407	440.2700323
Total	493	212,803	231.6696663	Total	382	212,053	180.1436433
Male (years)							
0–9	61	28,849	211.445804	0–9	76	28,783	264.0447486
10–19	28	28,609	97.87129924	10–19	22	28,558	77.03620702
20–29	38	27,473	138.3176209	20–29	11	27,558	39.91581392
30–39	17	28,564	59.51547402	30–39	19	28,755	66.07546514
40–49	31	35,061	88.4173298	40–49	16	36,223	44.17083069
50–59	18	37,347	48.1966423	50–59	18	36,781	48.93831054
60–69	26	34,063	76.3291548	60–69	20	33,741	59.27506594
70–79	15	21,256	70.56831012	70–79	18	20,498	87.81344521
80–89	13	9,616	135.1913478	80–89	5	9,279	53.88511693
90+	2	1,394	143.472023	90+	2	1,309	152.7883881
Total	249	194,774	127.8404715	Total	207	194,144	106.6218889

## Discussion

Methodologically, there are a number of ways of examining hospital admissions, i.e. the use of the health service, in the population. To be specific, we could look historically at the trends by day of the week, by month, by season or by year. We could also examine geographically by hospital, by city, by region or by country. Mathematically, we could estimate by number, by rate or by standardisation. Politically, we could assess by practice, by policy or by reform. For example, respiratory admissions declined accompanying an increase in smoke-free areas or with the introduction of immunisation [29–33]. Understanding the use of the health service in the bigger picture is critical for health service providers and policy makers to effectively re-allocate medical and social resources (from prevention to rehabilitation) respectively. The targeted at-risk population may shift following the change in investment in health and nursing programs and the subsequent risk contributor profile (biologically or non-biologically). Therefore, the performance review of such ought to be documented regularly, preferably annually.

### Strengths and limitations

The present study has a few strengths. First, the data are from recent years. Therefore, the results provide information on recent health policy use. Second, the study period covers full calendar years. In addition, the population size was estimated on a yearly basis. Therefore, selection bias could be avoided in the presentation of trends and the estimation of rates could be more accurate than using the population census from a single year. However, mis-classification may not be completely avoidable [34, 35]. Third, this is the first HES study looking at the use of the health service in respiratory disease from the Northumbria area, which is free from central governmental control. However, there are also a few limitations that cannot be ignored. First, it was not possible to link with population surveys to understand patient risk contributor profiles, whether biological or non-biological. However, the entire study focus was to investigate if and how different age groups could present any change in health service use in recent years. Second, only two genders were identified. In other words, transgender was not properly coded. Therefore, no results on transgender people could be obtained (more details via http://www.ons.gov.uk/ons/about-ons/business-transparency/freedom-of-information/what-can-i-request/previous-foi-requests/health-and-social-care/transgender-population-figures/index.html). Third, some coding errors might not be 100% avoidable, which would affect the estimates. Taken together, future studies retaining the strengths and overcoming the limitations mentioned above to continuously monitor and document such clinical evidence from the local setting to the national setting would be recommended.

### Research, practice and policy implications

From 2013 to 2014, there has been unchanged use of health service utilisation with regard to common respiratory diseases, except for asthma. Respiratory disease is a common condition that has a large and negative impact on quality of life and life expectancy, with high financial costs. To direct future research, local health policy and guidelines could benefit from annual clinical records on health service use for respiratory diseases. From the practice and policy perspectives, re-organising and re-diverting funding to improve population health on a yearly basis, including improving the role of health and nursing professionals in reducing the burden of rehabilitation and raising public awareness, attitude and knowledge may serve the changing need in local areas.
